# Suicides

**Published:** 1875-09

**Authors:** 


### SUICIDES.

Deaths by suicide appear to be largely on the increase. Very slight cause seems to be ample to induce self-taking-off. One man destroys himself because of unhappy domestic life; another because he has no domestic life at all. One takes Paris green because he has no work to do; another swallows morphine because his brother, as he supposes, pays other employees more promptly than he pays his own kinsman.

For the cowardly act of suicide, there is usually little excuse and slight palliation. We question if even the recent self-inflicted death of the Baroness Macedo, of Paris, could be deemed other than cowardly. She was about sixty years of age, and was the widow of a Portuguese Admiral, the Baron de Macedo. She possessed a handsome fortune, and went a great deal into the best society of Paris, mingling especially with the diplomatic set. For some years past she has been suffering from a cancerous tumor, to relieve which she had recently undergone a painful and dangerous operation. The operation had failed, and her physicians had informed her that it would be necessary for her to undergo another. As she had suffered terribly, she was heard to say that she would rather die than recommence. A few weeks ago her *femme de chambre*, on entering her bed-room in the morning, found the room full of charcoal gas, and Madame de Macedo extended insensible on the bed. She was revived with difficulty, and on coming to her senses scolded her servant roundly, saying that she had merely been asleep. The other day she gave her servants permission to go out for the afternoon, saying that she had received an invitation to go out driving and would not return till very late. When the servants got back they found Madame de Macedo lying a corpse on a feather bed in one of the corridors of her suite of apartments. Around her had been placed a number of furnaces, each of which had been filled with charcoal, and beside her lay an empty bottle labeled "ether." Medical assistance was at once summoned, but the physicians could only state the fact that she had been dead for more than an hour.

---

SOCIETY DISSIPATION.—It is sad to be compelled to say it, yet 'tis true that the faces of so many of the young ladies in New York society are ugly before their time. Months of increasing dissipation, round dances, late suppers, nights turned into day, all tell their story in the havoc they make with the fresh tints and outlines of youth and in the glance of innocence which should be the young soul's inalienable birthright. There are young women under twenty-five, the daughters of parents high in position, young ladies who flourish in every society column as "belles," of course "elegant, accomplished and beautiful," who walk the streets rouged to their eyes, with close mask veils, through which no one can trace the ravages that a false life—not time—has made upon them, but through whose thin meshes they look forth with bold, hard, defiant gaze, with lifted eyelids that never droop beneath the most prolonged or equivocal stare.

---

INCOMPATIBLE.—Do not give the two tonics—iron and quinine—together in wine, or in any other menstruum. United they neutralize each other and form a first-class article of ink.

